# The impact of *in vitro* cultivation on the natural life cycle of the tick-borne relapsing fever spirochete *Borrelia turicatae*

**DOI:** 10.1371/journal.pone.0239089

**Published:** 2020-10-12

**Authors:** Aparna Krishnavajhala, Brittany A. Armstrong, Job E. Lopez

**Affiliations:** 1 Department of Pediatrics, National School of Tropical Medicine, Baylor College Medicine, Houston, Texas, United States of America; 2 Department of Molecular Virology and Microbiology, Baylor College of Medicine, Houston, Texas, United States of America; University of Kentucky College of Medicine, UNITED STATES

## Abstract

Tick-borne relapsing fever is an infectious disease caused by *Borrelia* species and are primarily transmitted by *Ornithodoros* ticks. Prior work indicated that *in vitro* cultivated spirochetes remain infectious to mice by needle inoculation; however, the impact of laboratory propagation on the pathogens natural life cycle has not been determined. Our current study assessed the effect of serial cultivation on the natural tick-mammalian transmission cycle. First, we evaluated genomic DNA profiles from *B*. *turicatae* grown to 30, 60, 120, and 300 generations, and these spirochetes were used to needle inoculate mice. Uninfected nymphal ticks were fed on these mice and acquisition, transstadial maintenance, and subsequent transmission after tick bite was determined. Infection frequencies in mice that were fed upon by ticks colonized with *B*. *turicatae* grown to 30, 60, and 120 generations were 100%, 100%, and 30%, respectively. Successful infection of mice by tick feeding was not detected after 120 generations. Quantifying *B*. *turicatae* in tick tissues indicated that by 300 generations they no longer colonized the vector. The results indicate that *in vitro* cultivation significantly affects the establishment of tick colonization and murine infection. This work provides a foundation for the identification of essential genetic elements in the tick-mammalian infectious cycle.

## Introduction

Relapsing fever (RF) is a global and emerging vector-borne disease caused by spirochetes in the genus *Borrelia*. The pathogens are transmitted by human body lice, ixodid, and argasid ticks [1,2], and the disease is particularly burdensome on the impoverished. Clinical manifestation includes high fever, neurological symptoms, nausea, vomiting, preterm labor, and miscarriage [1]. As a neglected disease, there are several knowledge gaps regarding pathogenesis and the overall genomic stability of the spirochetes during laboratory cultivation.

The impact of genomic instability on the tick-mammalian transmission cycle of vector borne spirochetes has been demonstrated in Lyme disease (LD) causing pathogens [3,4]. The genome of LD causing spirochetes is complex containing between seven and 23 linear and nine circular plasmids [5,6]. Schwan et al. demonstrated that subculturing LD spirochetes resulted in a loss of a 7.6 and 22 kb plasmid, and this was associated with pathogen attenuation in mice [4]. Early studies by Kelly in the RF spirochete, *Borrelia hermsii*, reported that *in vitro* propagation after eight months (~326 generations) did not affect the pathogen’s infectivity in mice after needle inoculation [7].

Through recent efforts, the genomic organization and stability of RF spirochetes is gaining clarity. In general, these pathogens contain a linear chromosome and five to 10 linear and circular plasmids [8–10]. Work in *B*. *hermsii* and *Borrelia turicatae* demonstrated that while plasmids were retained during *in vitro* cultivation, segmental rearrangements and loss of DNA portions were observed by 520 generations in laboratory culture [11]. However, both species remained infectious in mice by needle inoculation after prolonged cultivation. While the studies provided insight into biological differences between LD and RF spirochetes, the consequence of prolonged *in vitro* cultivation on the tick-mammalian transmission cycle of RF spirochetes is unknown.

The infectious cycle of argasid-borne RF spirochetes requires adaptation to three environments, the tick midgut and salivary glands, and vertebrate host. Within the blood of the mammalian host, RF *Borrelia* species can reach between 1 x 10^4^ to 1 x 10^8^ spirochetes per ml of blood. Moreover, the pathogens up-regulate genes involved with antigenic variation to facilitate the escape from the host antibody response [12]. The dynamics between the host antibody response and antigenic variation can continue for several months, providing multiple opportunities for the acquisition of spirochetes by uninfected ticks. During an infectious bloodmeal, RF spirochetes enter the midgut, and in the following weeks a population migrates and colonizes the salivary glands. Salivary gland colonization is important because transmission occurs within seconds of tick bite [13], and this population of RF spirochetes is preadapted for establishing early mammalian infection. Given the dynamics of tick colonization and transmission, an improved understanding of the outcomes of *in vitro* propagation on the pathogen’s natural life cycle is critical.

In this study, *B*. *turicatae* was continuously grown for 300 generations and evaluated in the tick-mammalian transmission cycle. We analyzed plasmid profiles of *B*. *turicatae* cultured to 30, 60, 120, and 300 generations (g30, g60, g120, and g300) by pulse-field electrophoresis. We confirmed the infectivity of g30, g60, g120, and g300 by needle inoculation using a lower inoculum compared to previous work [11] and observed similar infection rates. To assess the natural transmission cycle of the *B*. *turicatae* g30–g300, nymphal acquisition was performed by feeding ticks on needle inoculated mice. After molting, these cohorts of ticks were fed again on naïve mice and the establishment of murine infection was determined. Since midgut and salivary glands colonization is essential in the pathogen’s life cycle, *B*. *turicatae* densities were also quantified in these tissues. This work signifies the impact of *in vitro* propagation of the natural life cycle of RF spirochetes and sets a foundation toward refined genetic studies for the identification of genes that are essential in tick-mammalian transmission cycle.

## Materials and methods

### Ethical statement

All performed work and animal husbandry was in accordance to the United States Public Health Service policy on Humane Care and Use of Laboratory Animals and the Guide for the Care and Use of Laboratory Animals. Murine studies were approved by the Baylor College of Medicine (BCM) Institutional Animal Care and Use Committee (protocols AN6563 and AN6580).

### Bacterial isolate and pulse-field electrophoresis

A polyclonal population of the 91E135 isolate of *B*. *turicatae* was used in this study [10], and spirochetes were cultured in modified Barbour-Stoenner-Kelly (mBSK) medium [14,15]. *B*. *turicatae* was grown at 35°C in 8 mL polystyrene tubes (Corning, New York, USA) to approximately 1 x 10^7^ bacteria per ml and every ~2 days, 50 μl were passaged into 4 mL of fresh mBSK medium that had been warmed to 35°C. Since the stationary cultures had about 4 x 10^7^ spirochetes per ml, the 50 μl used to inoculate fresh medium contained ~5 x 10^5^ spirochetes. Within 48 hours we quantified ~1 x 10^7^ spirochetes per ml in 4 mL of mBSK. Thus, we calculated ~6 generations (doublings) per passage. The number of *B*. *turicatae* generations used in this study were designated as g30, g60, g120, and g300. *B*. *turicatae* grown to 30 generations was equivalent to passaging the spirochetes five times after the original isolation, and this was the lowest passage we had in the laboratory. Genomic DNA (gDNA) from *B*. *turicatae* grown between 30 to 300 generations was isolated by phenol-chloroform extraction, and plasmid profiles were evaluated by pulse-field agarose gel electrophoresis, as previously described [11,16].

### Murine infections by needle inoculations and tick bite

The infectivity of *B*. *turicatae* g30, g60, g120, and g300 was determined in mice. For animal studies, six to eight-week-old female Institute of Cancer Research (ICR) mice were used. These are an outbred strain maintained at BCM. Five animals were needle inoculated intraperitoneally with 1 x 10^3^ spirochetes grown to each generation. Blood samples were collected for 10 consecutive days and infection was determined as detailed below.

### Murine infections by tick bite

Tick colonies used in the study were *O*. *turicata* that originated from Texas. These ticks were laboratory reared offspring of uninfected adults, as previously described [17]. To infect *O*. *turicata*, mice were needle inoculated with g60, g120, or g300 spirochetes. Cohorts of ~50 second nymphal stage ticks were fed to repletion when bacterial densities were ~1 x 10^6^ spirochetes per ml of blood in the mice. After molting into the third nymphal stage, seven to 10 ticks were subsequently fed on naïve animals. Transmission studies were performed twice with five mice each time.

### Determination of murine infection by microscopy and quantitative PCR (qPCR)

Murine infections were evaluated, as previously described [13]. For microscopy, a drop of blood (~2 μl) was collected by tail nick, and 20 microscopic fields were scanned with a 20x dark field objective (Carl Zeiss Microscopy, Munich, Germany) for 10 consecutive days after the transmission feedings. At the same time, 2.5 μl of blood was collected into 47.5 μl of Lysis-Stabilization Buffer (Agilent, Santa Clara, California, USA) from each animal for quantification. Primers and a probe for the flagellin gene (*flaB*) were used for qPCR assays and the conditions used were previously described [13]. A standard curve was generated for qPCR using *in vitro* grown *B*. *turicatae* serially diluted from 1 x 10^7^ to 1 x 10^3^ spirochetes per ml.

### Immunoblotting

One month after needle inoculations and tick transmission feedings to mice, serum samples were collected after sacrificing the animals with an overdose inhalation of isoflurane and exsanguination by cardiac puncture. Seroconversion to *B*. *turicatae* protein lysates was determined, as previously reported [13]. Serum samples were diluted at 1:200 and the secondary molecule was protein G-HRP (Life Technologies, Carlsbad, CA, USA) diluted at 1:4,000. Serological reactivity to *B*. *turicatae* whole protein lysates was detected by chemiluminescence using ECL Western Blotting Detection Reagents (GE Health Care, Buckinghamshire, UK).

### qPCR of *O*. *turicata* midguts and salivary glands

Individual midguts and salivary glands were excised from fifth stage nymphal ticks, as previously described [18]. A given assay had 4 to 5 biological replicates per tissue. Each replicate consisted of midguts or salivary glands from five ticks, and gDNA was extracted using the DNeasy Blood and Tissue kit (Qiagen, Hilden, Germany), as previously described [19]. Quantification of *flaB* relative to *β-actin* was performed in duplex qPCR assays to detect *B*. *turicatae* DNA, as preciously described [19]. An unpaired samples t-test was performed using Graphpad Prism 7.04 to determine if there was a significant difference in *B*. *turicatae flaB* copies between midgut tissues of ticks colonized with different generations of spirochete. Similarly, statistical differences in *flaB* copies were determined between salivary gland tissues.

## Results

### Plasmid profiles of *B*. *turicatae* after prolonged cultivation

**Fig 1:**
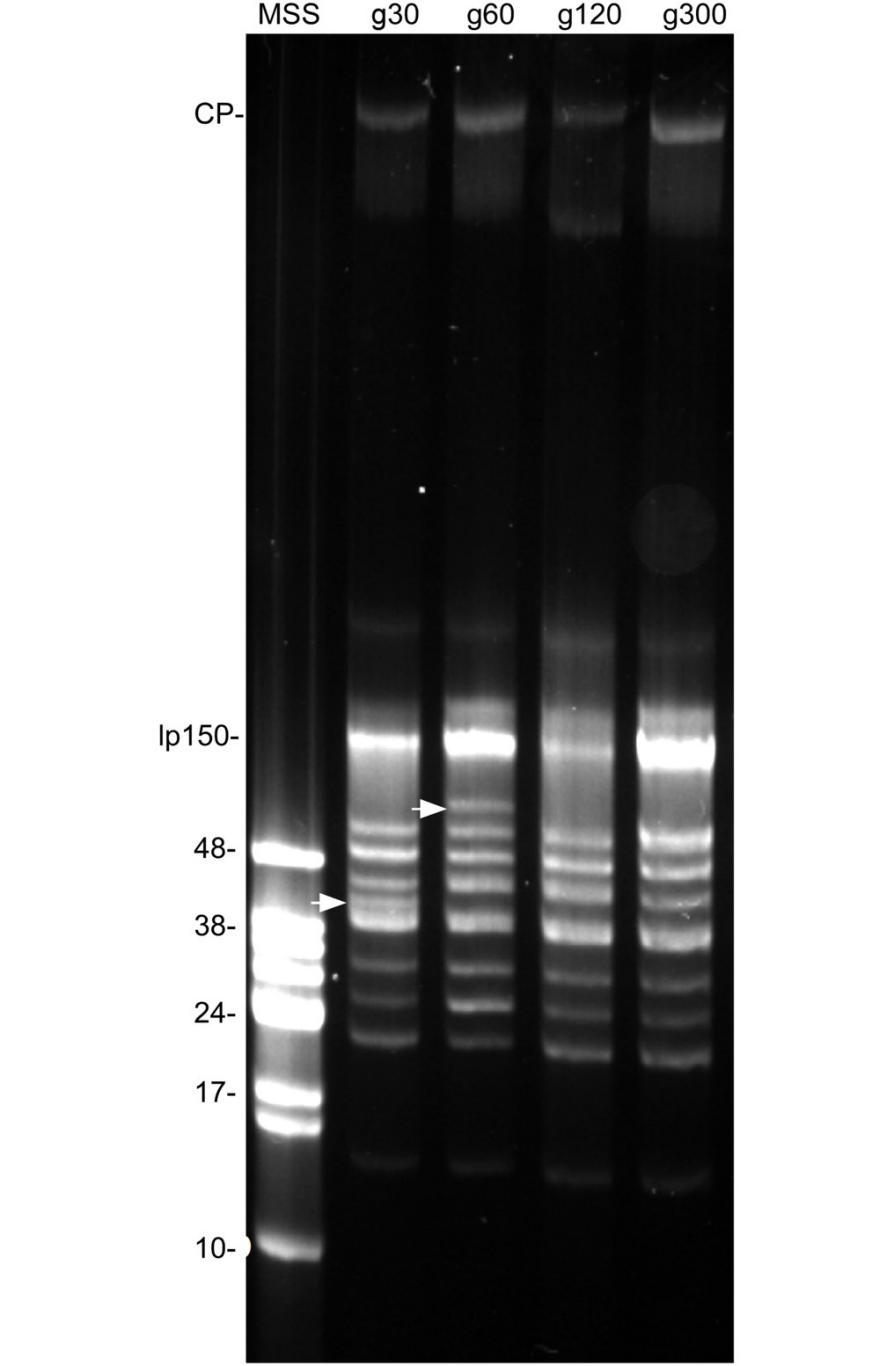
*B*. *turicatae* plasmid profiles from low to high generation passaged cultures. Genomic DNA was isolated from *B*. *turicatae* grown continuously *in vitro* to 30, 60, 120 and 300 generations. Arrows indicate unique plasmids at ~40 kb in g30 and ~80 kb in g60 cultures. Generation designations are shown above each lane (g30–g300). The molecular weight marker (MWM) shown on the left is Lambda monocut DNA. CP, circular plasmid; lp150, linear 150 kb megaplasmid.

To validate prior work [11], we evaluated genomic profiles of *B*. *turicatae* after prolonged cultivation. Pulse-field electrophoresis of *B*. *turicatae* indicated few changes in plasmid profiles. A ~40 kb linear plasmid was detected in spirochetes grown for 30 generations but was no longer detectable by pulse-field electrophoresis in the remaining generations (Fig 1). Interestingly, a ~80 kb plasmid was observed in DNA preparations from *B*. *turicatae* grown to 60 generations but was no longer detected in *B*. *turicatae* grown to 120 and 300 generations (Fig 1). As previously reported [11], these results suggested that plasmid rearrangements occurred during prolonged cultivation, and we further verified the infectious status of these spirochetes in mice.

### Murine infection after needle inoculating mice with *B*. *turicatae* g30, g60, g120, and g300

**Fig 2:**
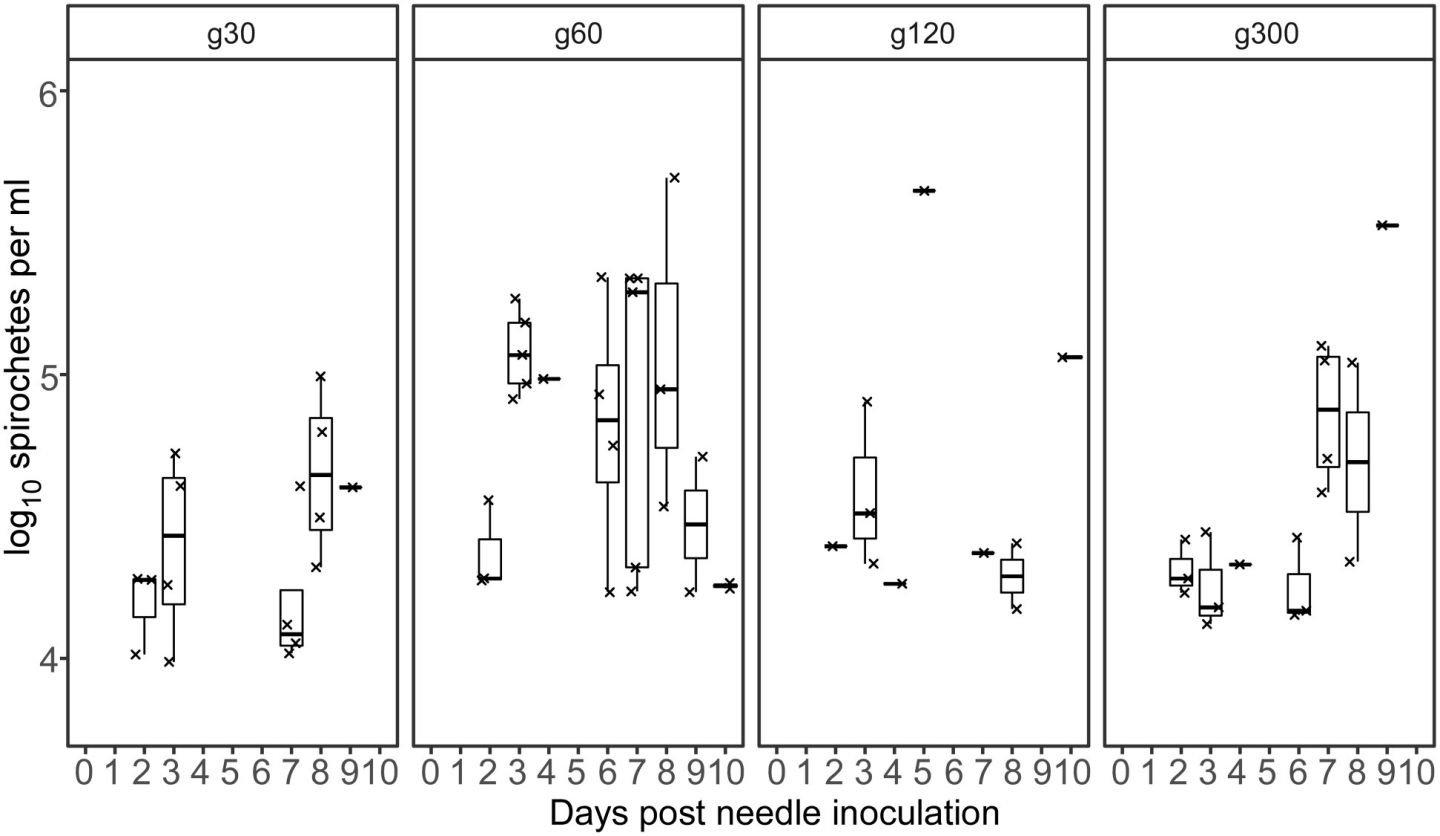
Quantification of spirochetes in infected murine blood after needle inoculations of *B*. *turicatae* grown to 30, 60, 120, and 300 generations. Each mark represents spirochete density from a single mouse infected by needle inoculation. Box and whisker plots show the median and the minimum and maximum spirochete densities per ml of blood from all infected animals. The generation of *B*. *turicatae* is shown above each box.

**Fig 3:**
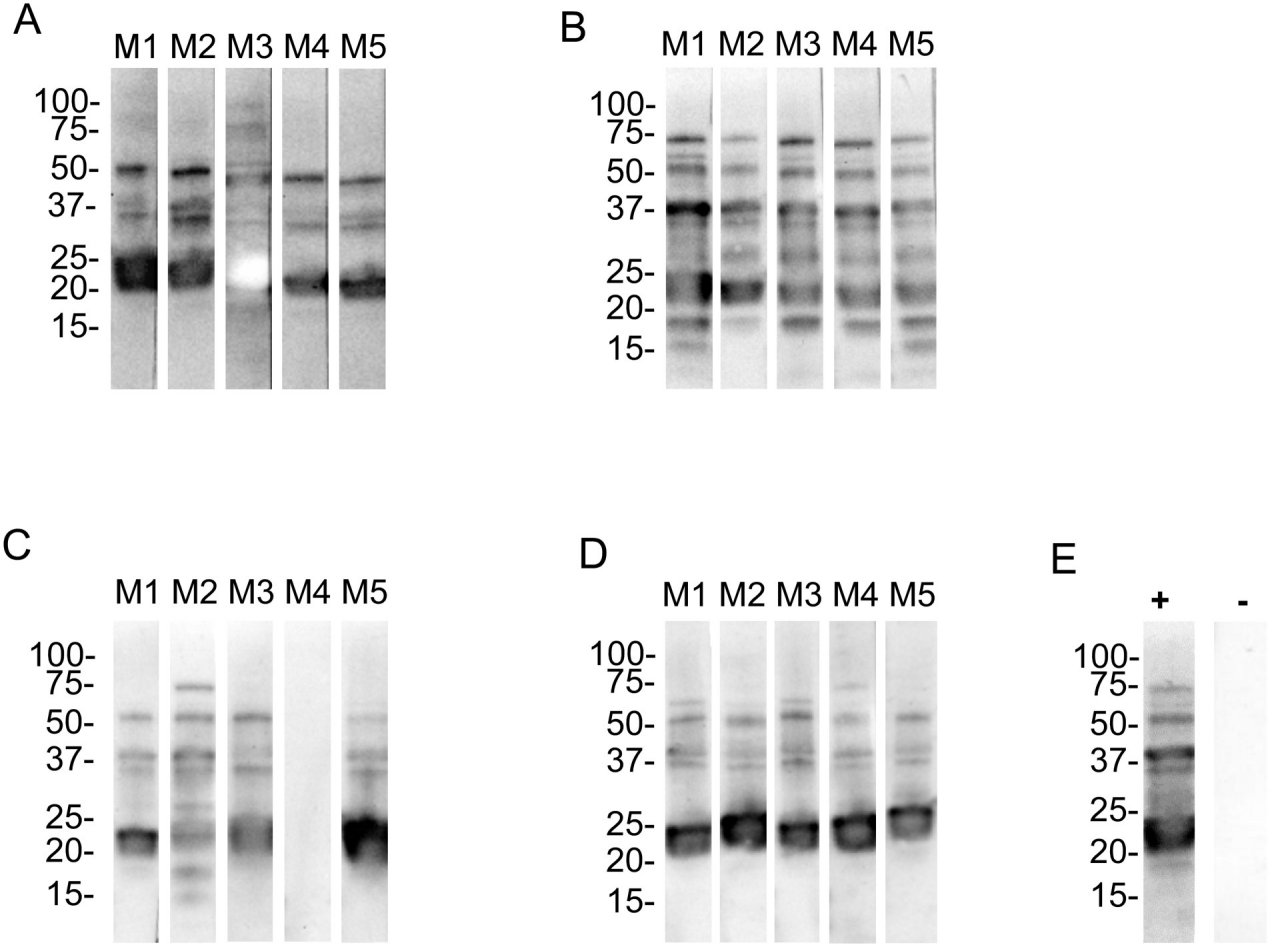
Serological responses to *B*. *turicatae* protein lysates from mice infected by needle inoculations. Immunoblots were probed with serum samples from mice inoculated with *B*. *turicatae* g30 (A), g60 (B), g120 (C), g300 (D). A positive and negative control serum sample was used from infected (+) and uninfected (-) mice, respectively (E). Molecular masses are indicated in kilodaltons on the left of each immunoblot.

The effect of long-term *in vitro* cultivation on the ability of *B*. *turicatae* to infect mice was previously studied using a high inoculum (1 x 10^5^ spirochetes) [11]. Consequently, we determined whether a lower dose of bacteria would establish infection. Except for one mouse inoculated with g120 spirochetes, bacteria were detected by qPCR in all the other animals inoculated with 1 x 10^3^ *B*. *turicatae* g30, g60, g120 and g300 (Fig 2 and Table 1). Regardless of the generation used to infect mice, spirochetes were detected in the blood by qPCR two days after inoculation. Additionally, infection was further confirmed by evaluating seroconversion to *B*. *turicatae* protein lysates one month after needle inoculation (Fig 3). These results indicated that at a low inoculum, *B*. *turicatae* is infectious by needle inoculation regardless of the generation.

**Table 1:** Murine infection frequencies after needle inoculation and tick transmission with *B*. *turicatae*.

*B*. *turicatae* generations	No. of mice positive by microscopy	No. of mice positive by immunoblotting
Needle inoculation		
g30^a^	4/5	5/5
g60^a^	5/5	5/5
g120^a^	3/5	4/5
g300^a^	5/5	5/5
Tick bite		
g60^b^	5/5	10/10
g120^b^	1/10	3/10
g300^b^	0/10	0/10

^a^ The inoculum was 1 x 10^3^ spirochetes.

^b^ Seven to 10 ticks were fed on each animal.

### Evaluation of the tick—mammalian infectious cycle using *B*. *turicatae* grown from 60 to 300 generations

**Fig 4:**
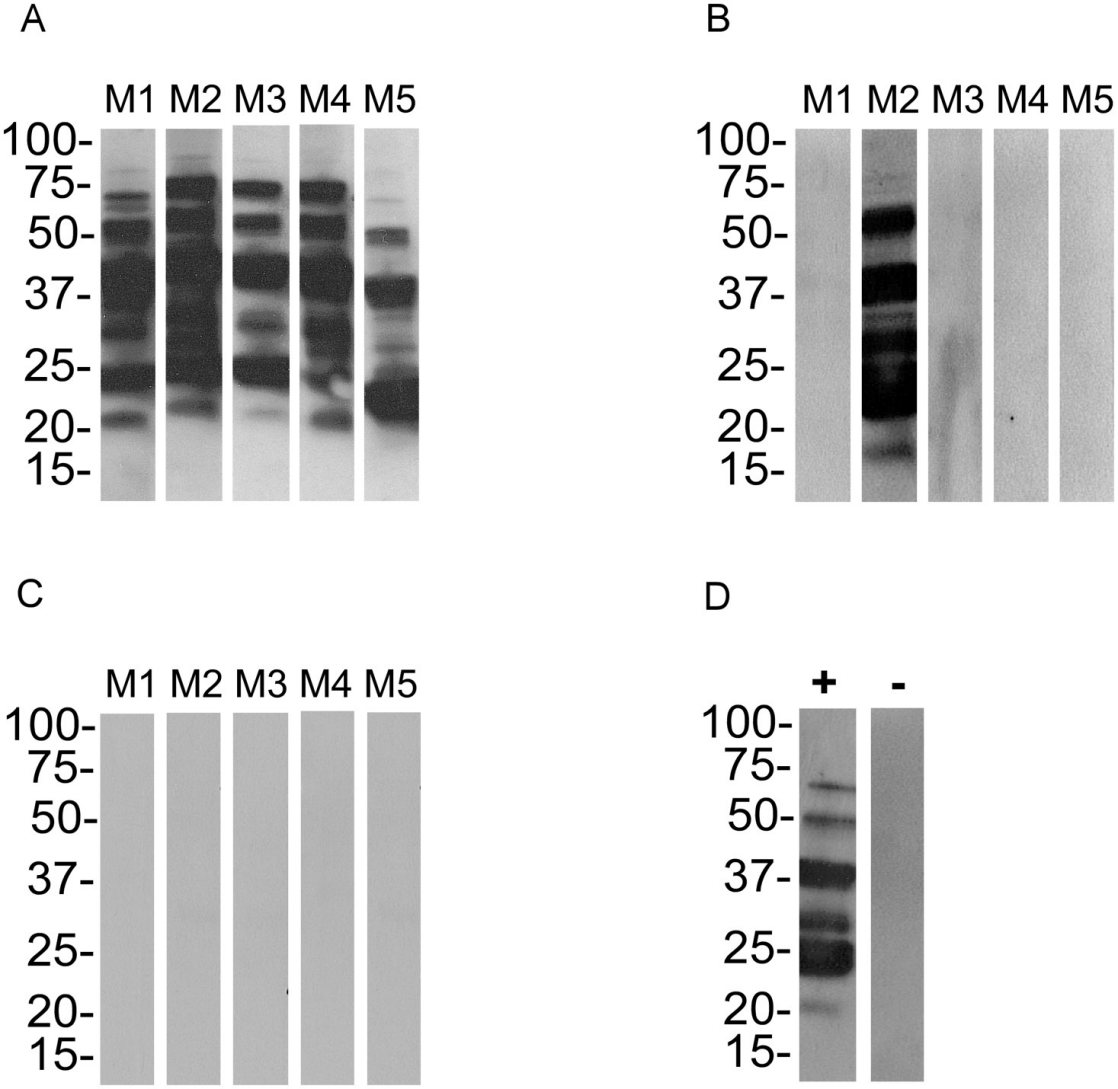
Serological responses of mice following transmission from ticks originally infected with *B*. *turicatae* grown to 60, 120, and 300 generations. Groups of five mice were used for tick transmissions and this was repeated for a total of 10 animals. Shown are immunoblots from one group of five mice infected with *B*. *turicatae* grown to 60 (A), 120 (B) and 300 (C) generations. Immunoblots using positive (+) and negative (-) control serum samples are shown and originate from *B*. *turicatae* infected and uninfected mice, respectively (D). Molecular masses are indicated on the left of each immunoblot in kilodaltons.

Since the infectious status of *B*. *turicatae* g30 by tick bite is well established [13,17–19], the remaining studies were performed using spirochetes grown to 60 generations and beyond. We infected mice by needle inoculation and once spirochetes attained ~1 x 10^6^ bacteria per ml of mouse blood, 50 third stage nymphs successfully fed to repletion. After molting, assessing tick transmission to naïve mice indicated that *B*. *turicatae* g60 remained infectious by tick bite (Table 1). Spirochetes were visualized in the blood by microscopy within four days after tick feeding. Furthermore, immunoblotting using serum samples from animals indirectly confirmed infection by detecting seroconversion to *B*. *turicatae* protein lysates (Fig 4 and Table 1).

An assessment of *B*. *turicatae* g120 by tick transmission indicated that the spirochetes were attenuated. Spirochetes were detected in one of 10 mice by dark field microscopy while three of the 10 animals seroconverted to *B*. *turicatae* protein lysates. By the 300^th^ generation, *B*. *turicatae* was no longer detectable by microscopy and none of the animals seroconverted, indicating that *B*. *turicatae* failed to establish an infection in these animals.

**Fig 5:**
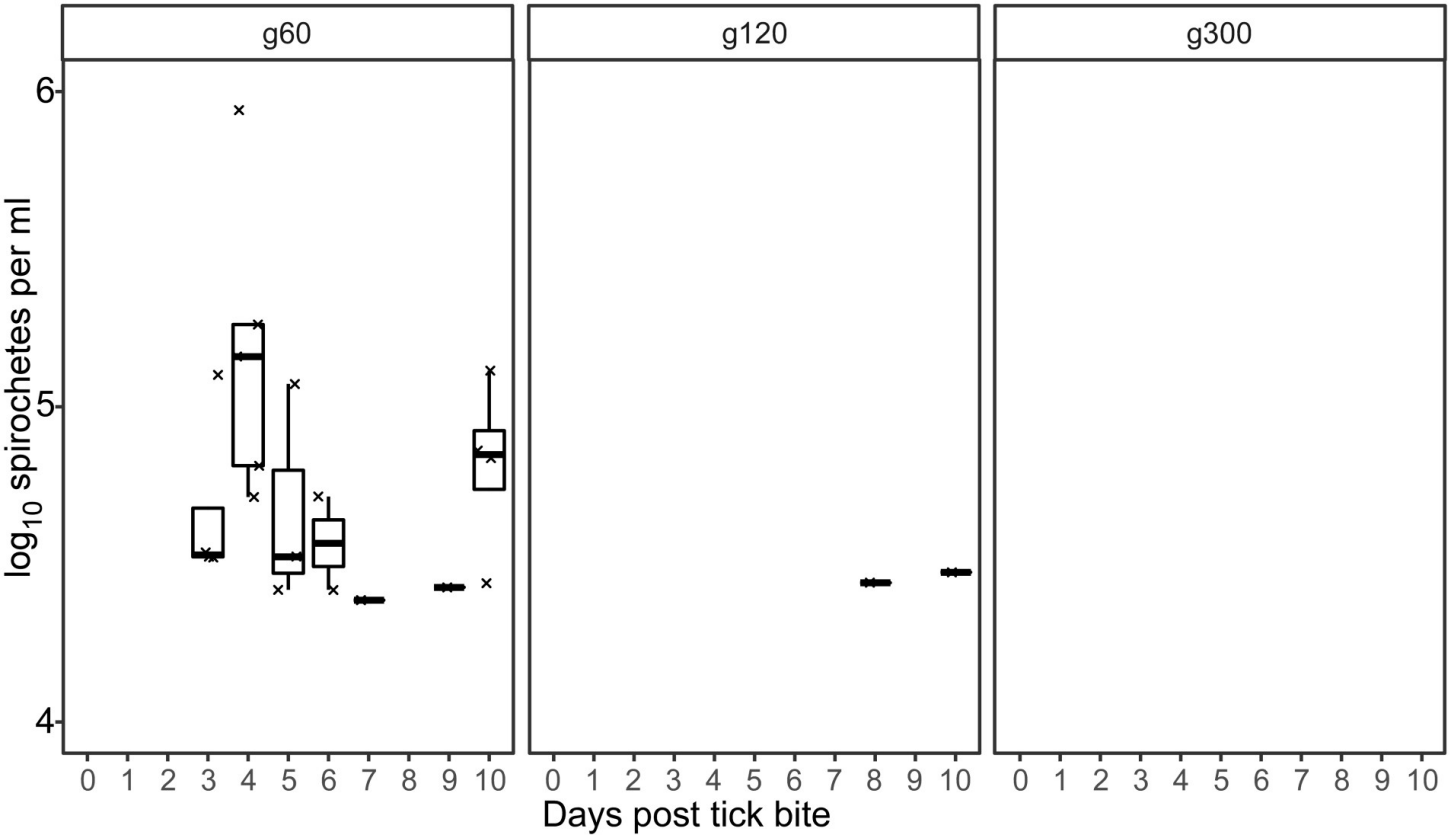
Quantification of spirochetes in murine blood following transmission from ticks originally infected with *B*. *turicatae* grown to 60, 120, and 300 generations. Each mark represents spirochete density from a single mouse infected by tick bite. Box and whisker plots show the median and the minimum and maximum spirochete densities per ml of blood from all infected animals. Each generation of *B*. *turicatae* is shown above each box.

We further quantified murine infection by qPCR to determine spirochete densities in the blood. qPCR analysis detected *B*. *turicatae* g60 DNA within three days after tick transmission, and all animals relapsed by 10 days (Fig 5). Spirochete densities in the blood ranged from 1 x 10^4^ to 1 x 10^6^ spirochetes per ml (Fig 5). These findings indicated that growing *B*. *turicatae* to 60 generations did not affect the spirochetes ability to establish and maintain an infection in mice after tick challenge.

One animal that was fed upon by ticks colonized with *B*. *turicatae* g120 became spirochetemic eight days after the transmission bloodmeal and relapsed (Fig 5). *B*. *turicatae* attained 1 x 10^4^ spirochetes per ml of blood in the mouse. The remaining animals were negative by qPCR. These results indicated that by 120 generations of continuous *in vitro* propagation, an attenuated phenotype was observed for *B*. *turicatae*.

### Quantification of *B*. *turicatae* g60 –g120 in tick midguts and salivary glands

Given the transmission findings after laboratory cultivation of *B*. *turicatae*, we determined whether the ticks used in the transmission feedings were colonized with spirochetes. To accomplish this, we developed a duplex qPCR assay to quantify spirochete densities in midgut and salivary gland tissues. A set of control qPCR experiments was performed targeting *B*. *turicatae flaB* and *O*. *turicata β-actin*. Primer and probe specificity and efficiency were assessed using pCR2.1::*flaB* and pCR2.1::*β-actin* plasmids. This indicated the specificity of the primers and we did not detect nonspecific binding. Furthermore, both primer sets were evaluated with each plasmid, which confirmed the absence of primer inhibition. qPCR assays using individual primer and probe sets against gDNA from *B*. *turicatae* and uninfected ticks further validated the specificity of the primers for their respective genes. Lastly, to generate standard curves, duplex qPCR assays were performed using 1 x 10^5^ to 1 x 10^1^ copies of pCR2.1::*flaB* and pCR2.1::*β-actin* plasmids, which indicated the compatibility of primer and probe sets (S1 Fig).

**Fig 6:**
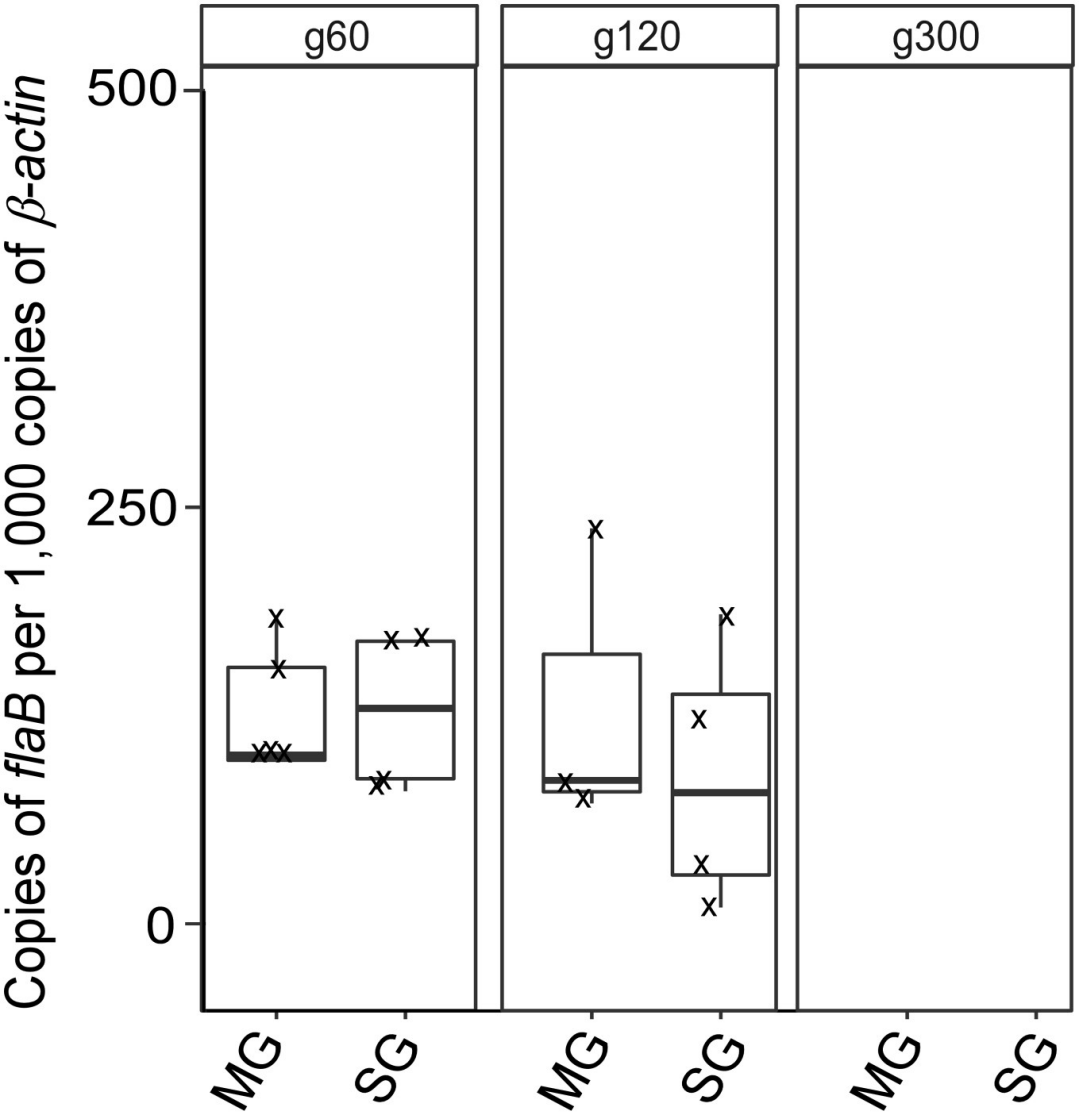
Quantification of *B*. *turicatae* DNA in ticks. Each midgut (MG) and salivary gland (SG) sample is a pool of respective tissues from five ticks, and at least four samples were evaluated per group. The generation of *B*. *turicatae* used to infect ticks is shown at the top of the image (g60–g300). Copies of *flaB* per 1000 copies of *β-actin* are also shown.

qPCR assays using gDNA from the midguts and salivary glands of flat ticks that were used in the transmission studies indicated that cohorts were similarly colonized with g60 and g120 spirochetes (Fig 6). There was no significant difference in copies of *B*. *turicatae flaB* detected in the midgut and salivary glands between the sample sets. However, *B*. *turicatae* g300 were undetectable by qPCR in both midgut and salivary gland samples (Fig 6). These findings demonstrated that prolonged *in vitro* cultivation impacted tick colonization and transmission.

## Discussion

This report begins to investigate the effect of *in vitro* propagation on the tick-mammalian transmission cycle of *B*. *turicatae*. Like prior work, we observed plasmid rearrangements but each generation of *B*. *turicatae* that was tested remained infectious in mice by needle inoculation [11]. While we evaluated a lower inoculum compared to prior work [11], mice still became infected regardless of the generation used. Interestingly, we observed that continuous laboratory cultivation disrupted the pathogen’s ability to colonize *O*. *turicata* and establish mammalian infection by tick transmission.

Genomic plasticity was previously observed for RF spirochetes, but this did not alter the pathogen’s ability to infect mice after needle inoculation [11]. *B*. *hermsii* and *B*. *turicatae* were subcultured from 50 to 520 generations. Southern blot analysis of plasmid marker genes indicated that DNA deletions and rearrangements occurred [11]. For example, *resT*, which was localized to a ~53 kb linear plasmid in *B*. *turicatae* grown to 50 generations mapped to a ~60 kb linear plasmid after ~520 generations. Furthermore, in both species of RF spirochete the factor H binding protein gene (*fhbA*) was lost from the 150 kb linear megaplasmid by ~520 generations [11]. In our current study, plasmid mapping was not performed by Southern blot, but we still observed DNA rearrangements by pulse-field electrophoresis. While the rearrangements did not seem to impact the infectivity of *B*. *turicatae* g60 by tick bite, by 120 generations we observed a phenotype.

*B*. *turicatae* g120 colonized the salivary glands of *O*. *turicata* to similar densities as g60 spirochetes, but we observed an attenuated phenotype in the ability to infect mice after tick transmission. We hypothesize that this was due to genomic instability and the polyclonal nature of the spirochetes. During *in vitro* cultivation, the absence of immunological pressures on *B*. *turicatae* likely resulted in variants within the population losing genetic material that was essential for preadapting them for mammalian infection. This was supported by our findings that seven of 10 mice failed to become infected after tick bite even though spirochete DNA was detected in the salivary glands at similar copies compared to the infectious *B*. *turicatae* g60.

Our findings also suggest that RF spirochetes do not transiently migrate through the midgut to colonize salivary glands. In flat persistently infected *O*. *turicata*, two spirochete populations are detected, one in the midgut and the other in the salivary glands [19]. Interestingly, the midgut population can be detected in ticks starved over 18 months [19]; however, their role in pathogenesis remains unclear. This is because transmission occurs within seconds of tick attachment and it is the salivary gland population of spirochetes that enters the vertebrate host [13]. In our study, *B*. *turicatae* DNA was undetectable in the *O*. *turicata* midguts and salivary glands by 300 generations. We also performed these studies with *B*. *turicatae* grown to 450 and 600 generations and observed identical results. These findings suggest that prolonged laboratory cultivation of *B*. *turicatae* resulted in the loss of genetic material that was essential for two likely situations. First, the ability of *B*. *turicatae* to initially colonize the tick midgut and subsequently the salivary glands. Second, spirochetes lost the ability to be transstadially maintained through the molt.

In this study, a polyclonal population of *B*. *turicatae* was used to assess vector competence, and for this initial work there were advantages over using clonal populations. For example, *O*. *turicata* is not commercially available and generating enough ticks to screen multiple clones at each generation would have been challenging. Additionally, utilizing a polyclonal population for spirochetes grown to 120 generations was advantageous as we began to detect a phenotype. Alternatively, if clones would have been used, we could have erroneously concluded that *B*. *turicatae* was no longer infectious after 120 generations. However, by 120 generations the population of *B*. *turicatae* was a clear mixture of infectious and noninfectious spirochetes. Now that we understand at what point spirochetes become noninfectious, further studies will focus on clonal populations to identify the molecular constituents essential for vector competence.

The molecular mechanisms of vector colonization and the establishment of early mammalian infection are unclear. Whole genome sequencing of clonal *B*. *turicatae* grown to 30, 60, and 120 generations will likely identify genes that were lost or disrupted during serial cultivation. Moreover, future studies will sequence infectious and noninfectious clones of *B*. *turicatae* g120 to identify genes responsible for the establishment of early mammalian infection. Mechanistic studies are possible as these genes can be further characterized with the established genetic system for *B*. *turicatae* [18]. We also envision the identification of gene subsets essential for early midgut colonization of the tick and transstadial maintenance. These candidates will provide proteins to target with the goal of interrupting the tick-mammalian transmission cycle of RF spirochetes.

## Supporting information

S1 Fig Amplification plots of duplex qPCR standard curves. *flaB* and *β-actin* were cloned into PCR2.1 vectors and designated PCR 2.1::*flaB* and PCR2.1::*β-actin*. To generate standard curves of the assays, serial dilutions of each plasmid were used from 1 x 10^5^ to 1 x 10^1^ copies and duplex qPCR was performed. Log copy numbers of each plasmid (*flaB*, blue and *β-actin*, orange) are indicated on the x-axis and average Ct-values on the y-axis. The equation of a line and the R^2^ values are shown.
(PDF)

S1 Raw images (PDF)

S1 File The ARRIVE essential 10: Author checklist. (PDF)
