# Supplementary material for: The impact of in vitro cultivation on the natural life cycle of the tick-borne relapsing fever spirochete Borrelia turicatae
Source: PLoS One. 2020 Oct 12;15(10):e0239089. doi: 10.1371/journal.pone.0239089 (PMC7549772; doi:10.1371/journal.pone.0239089)
Supplement: S1 Fig — flaB and β-actin were cloned into PCR2.1 vectors and designated PCR 2.1::flaB and PCR2.1::β-actin. To generate standard curves of the assays, serial dilutions of each plasmid were used from 1 x 10^5^ to 1 x 101 copies and duplex qPCR was performed. Log copy numbers of each plasmid (flaB, blue and β-actin, orange) are indicated on the x-axis and average Ct-values on the y-axis. The equation of a line and the R2 values are shown. (PDF) [file pone.0239089.s001.pdf]

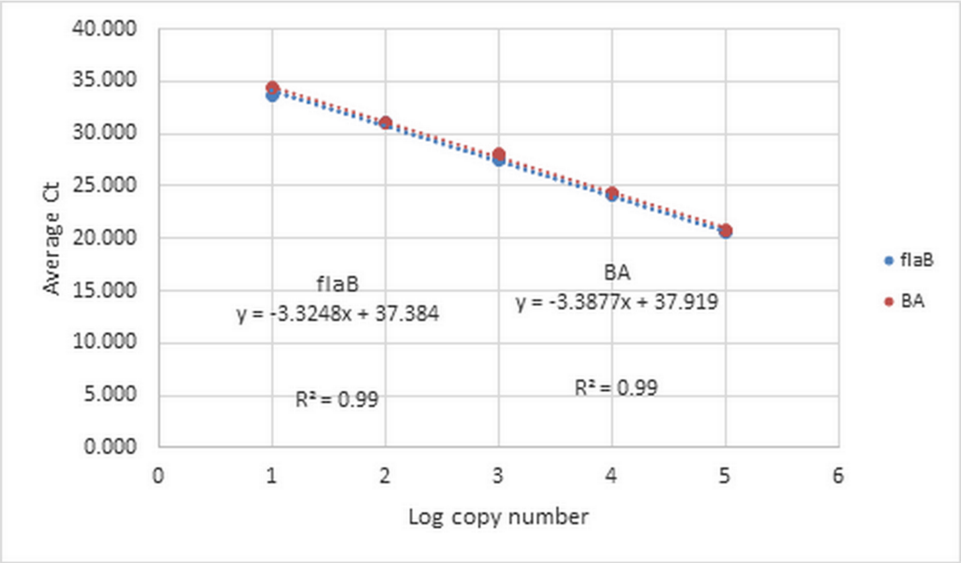

Supplementary Figure 1. **Amplification plots of duplex qPCR for standard curves.** Cloning of *flaB* and *β actin* into PCR 2.1 vector generated PCR2.1::*flaB* and PCR2.1::*β actin*. Serial dilutions of *flaB* and *β actin* plasmids from 1 X 10^5 to 1 X 10^1 copies were used to generate standard curves from duplex qPCR reaction. Log copy numbers of *flaB* and *β actin* plasmids are indicated on the x-axis and average Ct values on the y-axis; their equation and R^2 values are represented in blue and orange colors respectively
