# Supplementary material for: The impact of in vitro cultivation on the natural life cycle of the tick-borne relapsing fever spirochete Borrelia turicatae
Source: PLoS One. 2020 Oct 12;15(10):e0239089. doi: 10.1371/journal.pone.0239089 (PMC7549772; doi:10.1371/journal.pone.0239089)
Supplement: S1 Raw images — (PDF) [file pone.0239089.s002.pdf]

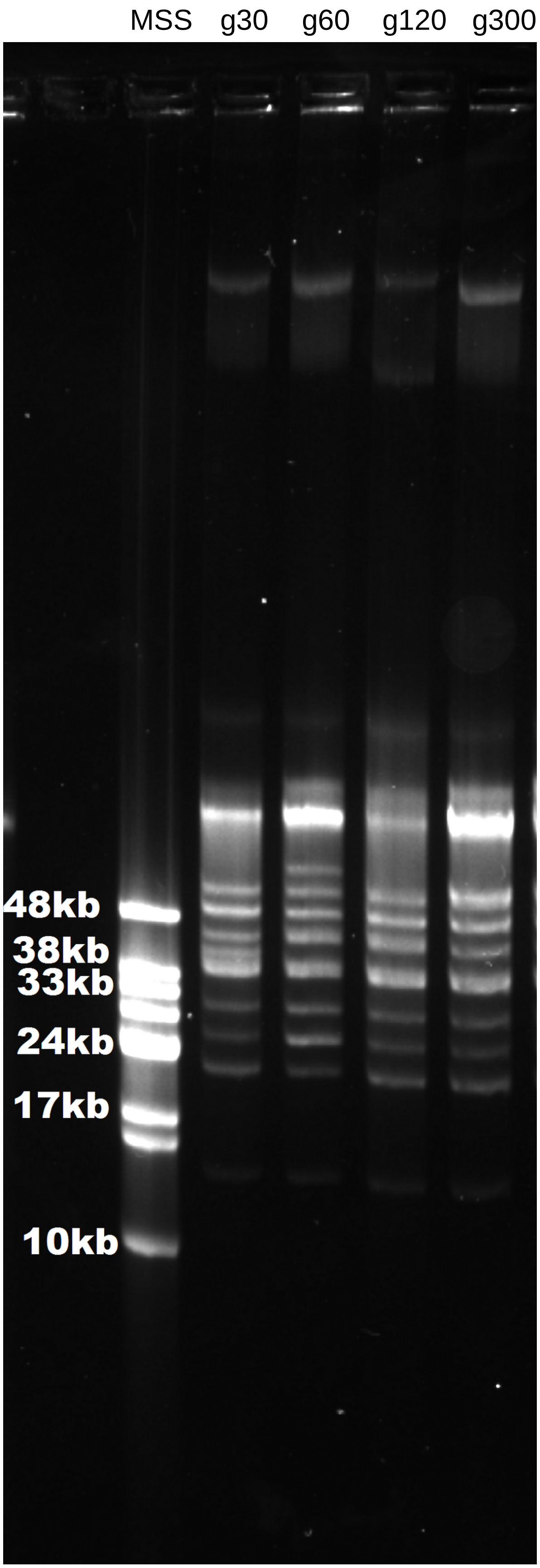

Figure 1 raw image. Genomic DNA from g30, g60, g120, and g300 is shown. Molecular size standards (MSS) are also shown.

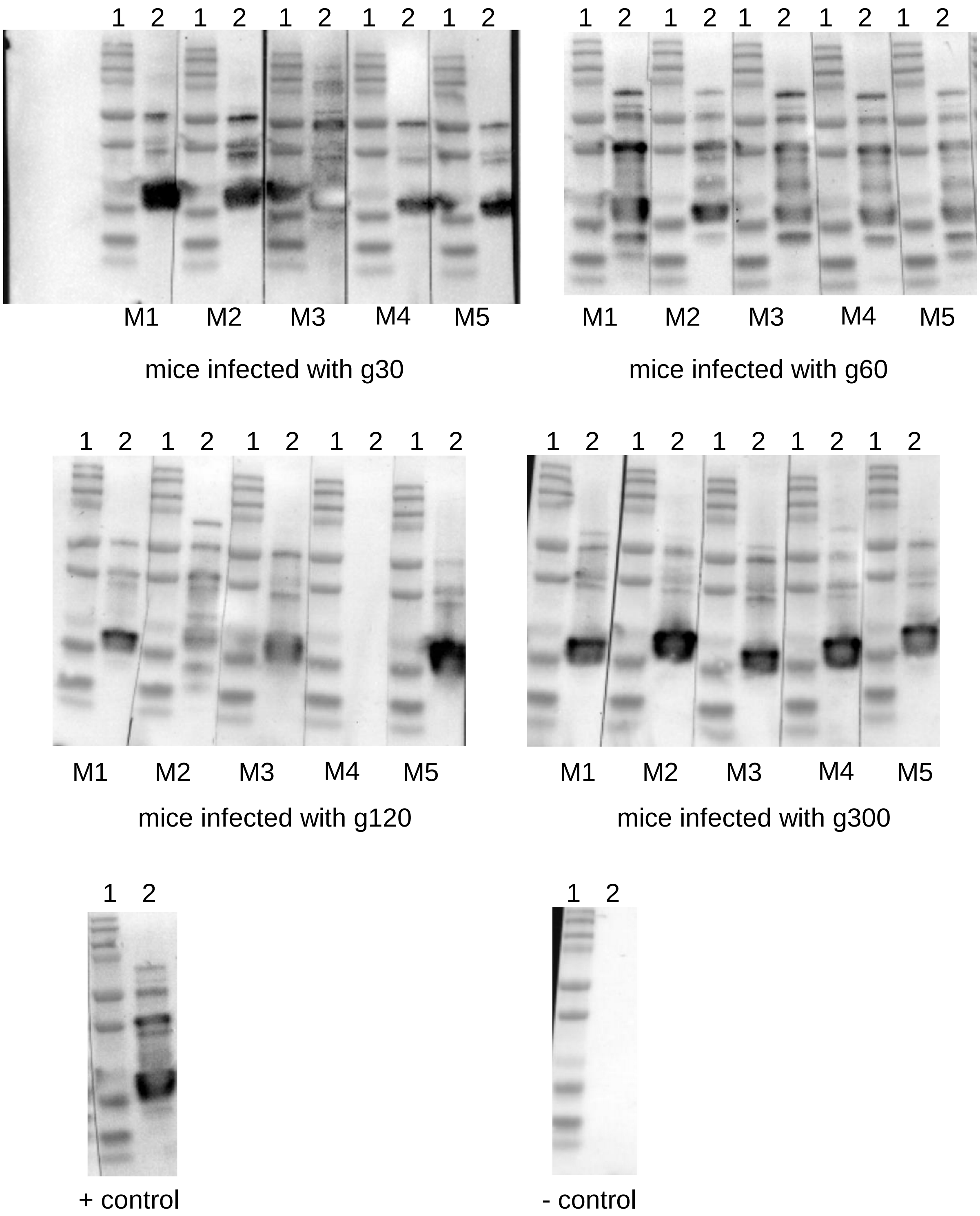

Figure 3 raw image. Lane 1 are the molecular weight markers that were overlaid for reference after developing the immunoblot. Lane 2 is B. turicatae protein lysate.

1 2 1 2 1 2 1 2 1 2

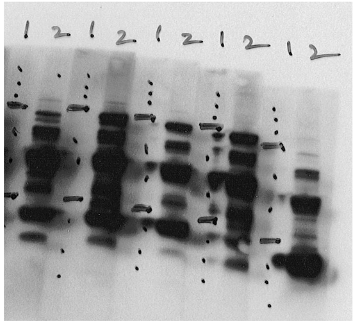

M1 M2 M3 M4 M5

mice infected with g60

1 2 1 2 1 2 1 2 1 2

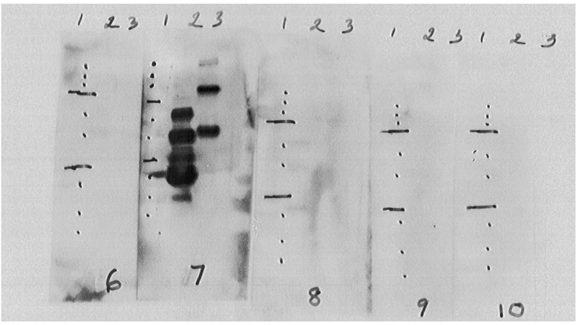

M1 M2 M3 M4 M5

mice infected with g120

1 2 1 2 1 2 1 2 1 2

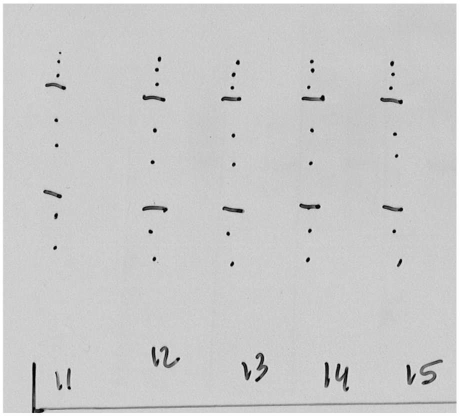

M1 M2 M3 M4 M5

mice infected with g300

Figure 4 raw images. A series of immunoblots were performed using serum samples from needle inoculated mice. Lane 1 is the molecular weight marker and lane 2 is B. turicatae protein lysate. Mouse number is shown below each immunoblot. Also, in immunoblots from mice infected with g120 there is a third lane. The antigen in that lane is a recombinant protein irrelevant to this study.
